# Prognostic factors for long-term outcomes of unilateral atrophic kidneys in adult patients: a single-center retrospective cohort study

**DOI:** 10.55730/1300-0144.6016

**Published:** 2025-04-28

**Authors:** Zülal İSTEMİHAN, Ahmet Burak DİRİM, Ali Rıza UÇAR, Ceren BAĞRIAÇIK, Gamze KEMEÇ, Şafak MİRİOĞLU, Seda ŞAFAK, Vafa SULEYMANOVA, Özgür Akın OTO, Ayşe Serra ARTAN, Alaattin YILDIZ, Aydın TÜRKMEN, Halil YAZICI

**Affiliations:** 1 Department of Internal Medicine, İstanbul Faculty of Medicine, İstanbul University, İstanbul, Turkiye; 2 Division of Nephrology, Department of Internal Medicine, İstanbul Faculty of Medicine, İstanbul University, İstanbul, Turkiye

**Keywords:** Chronic kidney disease, renal hypoplasia, unilateral atrophic kidney

## Abstract

**Background/aim:**

Unilateral atrophic kidney (UAK) could be related to many etiologies that can cause chronic kidney disease in adults. There is limited data on the long-term outcome of adults with UAK in the literature.

**Materials and methods:**

This study included 199 adult patients with UAK. The etiology, baseline clinical/laboratory, and radiological findings were evaluated. Composite primary outcomes (CPO) (chronic kidney disease stage 5 or doubling of serum creatinine) and secondary outcomes (new-onset proteinuria (>0.5 g/day or g/g) or >50% increase in proteinuria level according to baseline, mortality, and nephrectomy requirement during follow-up) were evaluated in 166 patients with at least 3 months of follow-up.

**Results:**

Of 199 patients, 57.3% were female. The mean age at presentation was 44.4 years. Right and left kidney atrophy rates were 51.8% and 48.2%, respectively. Among the known etiologies, the most common was chronic pyelonephritis (17.1%, n = 34). Of 166 patients, 19 had a CPO. Patients with CPO had higher rates of hypertension (p = 0.033), proteinuria (p < 0.001), and renal artery stenosis. Baseline systolic blood pressure (p = 0.004) and serum creatinine (p < 0.001) were higher, and baseline eGFR (p < 0.001) and serum albumin (p = 0.001) were lower in these patients than in patients without CPO. Multivariate logistic regression analysis showed that baseline creatinine (p < 0.001), serum albumin (p = 0.034), and renal artery stenosis (p = 0.015) were independent risk factors for CPO.

**Conclusion:**

Higher baseline serum creatinine and lower serum albumin levels were associated with poor renal prognosis in adult patients with UAK. Also, UAK due to renal artery stenosis might be associated with worse outcomes than UAK related to other etiologies.

## 1. Introduction

Unilateral atrophic kidneys could be related to congenital or acquired causes in adults. The etiological factors can be summarized as congenital renal hypoplasia or dysplasia, renal artery stenosis, chronic renal vein thrombosis, radiation nephritis, reflux nephropathy, papillary necrosis, renal tuberculosis, renal infarction, and chronic pyelonephritis [1,2].

There are many imaging methods for diagnosing renal atrophy, including ultrasonography (USG), computed tomography, magnetic resonance imaging, and scintigraphy. These imaging methods not only provide information about kidney dimensions but also show factors that could affect kidney size, such as vascular problems, the presence of stones, cysts, masses, infections, obstructions, and congenital anomalies in the kidney [3–5]. Besides radiographic methods, Tc-99m-DMSA (dimercaptosuccinic acid) scintigraphy, which is one of the radio nuclear studies, is an important radionuclear imaging method to calculate the contribution of each kidney to the total kidney function (separated/split/relative renal function) [6,7].

In terms of clinical findings, atrophic kidneys may cause hypertension, proteinuria, persistent flank pain, recurrent urinary tract infections, and chronic kidney disease. Sometimes patients require nephrectomy due to recurrent infections, nephrolithiasis, and uncontrolled hypertension, and these symptoms may improve after nephrectomy [8–10]. Glomerular hyperfiltration and glomerular hypertrophy due to the reduced renal mass, low nephron number, and decreased glomerular filtration surface area are the major culprits for the development of systemic hypertension, proteinuria, glomerulosclerosis, and progressive renal failure [11–13].

Although many pediatric studies regarding atrophic kidneys have been published, a limited number of publications that include the long-term outcomes of unilateral atrophic kidneys in adult patients are available [1,14,15]. In this retrospective single-center study, we investigated etiological and prognostic factors in adult patients with unilateral kidney atrophy. To the best of our knowledge, this is the largest adult cohort with unilateral atrophic kidney and long-term outcomes in the literature.

## 2. Materials and methods

In this single-center retrospective study, we included 199 patients with unilateral atrophic kidneys who were followed up at İstanbul University, İstanbul Medical Faculty Nephrology Department between 1994 and 2023. Kidneys with a craniocaudal length less than 10 cm measured by USG and/or kidneys with a difference of at least 30% between the right and left kidneys with Tc-99m-DMSA were considered atrophic kidneys. None of these patients had a baseline eGFR of <15 mL/min/1.73 m^2^. All patients’ creatinine levels were calculated using the Jaffe method during the follow-up period.

The etiology of the atrophic kidney (unknown, congenital, recurrent urinary tract infection, nephrolithiasis, vesicoureteral reflux, or renal artery stenosis), accompanying other urinary anomalies, baseline Tc-99m-DMSA kidney scintigraphy results, kidney sizes measured by USG, serum creatinine, serum albumin, and eGFR levels using the CKD-EPI equation, urinalysis, proteinuria levels (spot urine protein/creatinine ratio), low-density lipoprotein (LDL), high-density lipoprotein (HDL), total cholesterol and triglyceride levels, presence of concomitant diabetes mellitus (DM) or hypertension (HT), and use of angiotensin-converting enzyme (ACE-i) or angiotensin receptor blocker (ARB) treatments were extracted from patient files. Complications and kidney function were evaluated during the follow-up.

None of the patients included in the study used nephrotoxic drugs other than ACE-i or ARB. Likewise, there is no comorbidity other than DM or HT. Patients with contrast nephropathy that may affect nephrological survival, antibiotic use that may affect kidney functions, or heart failure were not included in the study.

The composite primary endpoint of our study was the doubling of serum creatinine levels (numerically, at least a 100% increase in creatinine level) or the development of chronic kidney disease (CKD) stage 5. An eGFR level of <15 mL/min/1.73 m^2^ at the last control was accepted as the definition of CKD stage 5. New-onset proteinuria (>0.5 g/day or g/g) or >50% increased rate of proteinuria level according to baseline, mortality, and nephrectomy requirement during follow-up were secondary outcomes. A total of 166 of 199 patients had at least 3 months of follow-up, and these 166 patients were included in the statistical analysis to evaluate the risk factors for outcomes.

Approval for this study was obtained from the Ethics Committee of İstanbul University Faculty of Medicine on 28.06.2019. The protocol number is 2019/731. All the applied procedures complied with the ethical standards of the Human Testing Committee of our institution and the Helsinki Declaration. Due to the study’s retrospective nature, the requirement for written informed consent was waived, and approval was received from the ethics committee of the İstanbul Faculty of Medicine.

### 2.1. Statistical analysis

In the descriptive statistics of the data, the mean, standard deviation, median (IQR 25–75), and frequency values were used. The distribution of variables was measured using the Kolmogorov-Smirnov test. The Mann-Whitney U test and independent samples t-test were used to analyze quantitative independent data. Paired sample t-tests and Wilcoxon tests were used to analyze the dependent quantitative data. The chi-square test was used in the analysis of independent qualitative data, and the Fisher test was used when the chi-square test conditions were not met. Variables with a p < 0.1 in the univariate analysis were included in the multivariate logistic regression analysis for predicting the odds ratios of variables for the primary composite outcome. The results were evaluated at a 95% confidence interval and significance level of p < 0.05. Data analysis was performed using SPSS version 26.0.

## 3. Results

### 3.1. Demographic and radiologic characteristics of the patient group

Of 199 patients, 57.3 % (n = 114) were female. The mean age at presentation was 44.4 ± 16.8 years. Of the patients, 66.8 % (n = 133) had hypertension, and 12.6% (n = 25) had diabetes mellitus. ACE-i or ARB treatment was used in 46.7% (n = 93) of the patients. The right and left kidney atrophy rates were 51.8% (n = 103) and 48.2% (n = 96), respectively. At admission, the mean kidney sizes of patients with right and left atrophic kidneys were 72.8 ± 17.9 and 73.6 ± 18.7 mm by USG, respectively. In addition, the nonatrophic kidney dimensions (right/left) were 117.9 ± 14.2 / 118.2 ± 14.9 mm. The mean separated kidney function percentages by Tc-99m-DMSA were 14.7 ± 10.6 % for right atrophic kidneys (n = 76) and 15.6% ± 12.1% for left atrophic kidneys (n = 87). Characteristics of 199 patients with unilateral atrophic kidneys are shown in Table 1.

In our study, 27 patients whose right kidney was not atrophic had kidney measurement control USG during follow-up. The first mean kidney size measurement of these patients was 118.0 ± 14.4 mm, and the last mean kidney size measurement was 117.9 ± 12.9 mm. 34 of our patients whose left kidney was not atrophic underwent kidney measurements during follow-up. The first mean kidney size measurement of these patients was 117.3 ± 14.5 mm, and the last mean kidney size measurement was 115.7 ± 15.6 mm.

The etiology could not be determined in 63.8 % (n = 127) of the patients (No clinical and radiological signs of nephrolithiasis, recurrent pyelonephritis, or renal artery stenosis. Congenital atrophic kidneys could not be ruled out). Among the known causes, the most common was chronic pyelonephritis (17.1%, n = 34). In addition, 9.5% (n = 19) of the patients had nephrolithiasis, 3.5% (n = 7) had vesicoureteral reflux (VUR), 3% (n = 6) had renal artery stenosis, 2% (n = 4) had congenital causes, and 1% (n = 2) had other causes (external pressure on the ureter and ureter ligation during intra-abdominal operation). The etiologies of atrophic kidneys are shown in Table 2. In addition, 5% of these patients had accompanying urinary-system anomalies. The anomalies are listed in Table 3.

### 3.2. Composite primary outcome

A total of 166 of 199 patients with at least three months of follow-up were included in the evaluation of the composite primary outcome. The demographic, laboratory, and clinical features of the 166 patients are provided in Table 4.

CKD stage 5 developed in 15 patients, and 13 patients had doubling of serum creatinine levels during follow-up (median follow-up time was 69.5 months, the mean follow-up time was 88.7 ± 66.7 [min 3–max 350] months). The composite primary outcome was observed in 19 of the 166 (11.4%) patients. The demographic, laboratory, and clinical features of patients with or without primary composite outcomes are shown in Table 5. Patients with a composite primary outcome had higher rates of hypertension (p = 0.033), renal artery stenosis (p = 0.021), baseline systolic blood pressure (p = 0.004), baseline proteinuria (p < 0.001), and baseline serum creatinine (p < 0.001). In addition, the serum albumin (p = 0.001) and baseline eGFR (p < 0.001) levels were lower in these patients.

### 3.3. Secondary outcomes

Mortality was observed during follow-up in six of 166 (3.6%) patients. In addition, 6 of the 166 (3.6%) patients required nephrectomy. A new-onset or >50% increase in proteinuria level according to baseline was observed in 21 (12.6%) of 166 patients. No statistical difference was observed in terms of secondary outcomes between patients with or without primary composite outcomes are shown in Table 6.

### 3.4. Complications during follow-up

Complications associated with unilateral atrophic kidney in 199 patients included recurrent urinary tract infections, nephrolithiasis, nephrectomy, doubling of serum creatinine, and CKD stage 5 development are shown in Table 7. The most common complication was recurrent urinary tract infections (25.6% of patients).

### 3.5. Logistic regression analysis for composite primary outcome

Variables with p < 0.1 in the univariate analysis, which were evaluated by multivariate binary enter logistic regression analysis for the composite primary outcome, were baseline systolic blood pressure, baseline diastolic blood pressure, baseline serum creatinine, baseline eGFR, baseline serum albumin, baseline proteinuria, presence of hypertension, and presence of renal artery stenosis. Correlations between these variables (Spearman or Pearson’s tests) were evaluated for collinearity before the establishment of the multivariate model. Also, multivariate logistic regression models were evaluated using a correlation matrix. If the coefficient values in the correlation matrix were >0.6 between two variables, one of these variables was chosen for multivariate analysis to establish the final model.

The final multivariate analysis model included baseline creatinine level, baseline serum albumin level, and the presence of renal artery stenosis. According to the model, baseline creatinine (p < 0.001), baseline serum albumin (p = 0.034), and the presence of renal artery stenosis (p = 0.015) were independent risk factors for the composite primary outcome are shown in Table 8. The explanatory coefficient of the multivariate analysis model was 92.2%. The correlation matrix of the model is presented in Table 9.

## 4. Discussion

Atrophic kidneys can cause hypertension, proteinuria, persistent flank pain, recurrent urinary tract infections, and kidney failure. Also, some patients might require nephrectomy [8–10]. In our study, 166 of 199 patients with at least three months of follow-up were included in the statistical analysis. 19 patients had a composite primary outcome. Fifteen developed end-stage renal disease, and 13 had doubled serum creatinine levels. Patients with composite primary outcomes showed higher rates of hypertension, proteinuria, and renal artery stenosis. Additionally, baseline systolic blood pressure and serum creatinine were higher, and baseline eGFR and serum albumin were lower in those patients than in those without the primary outcome. Multivariate logistic regression analysis showed that baseline creatinine and serum albumin levels and renal artery stenosis were independent risk factors.

It is a well-known fact that glomerular hyperfiltration and hypertrophy due to the decrease in renal mass, nephron number, and glomerular filtration surface area might cause systemic hypertension, proteinuria, glomerulosclerosis, and progressive renal failure [11–15]. In a study by Rugiu et al. of 22 patients with unilateral renal agenesis or nephrectomy, agenesis or nephrectomy was associated with the development of hypertension, proteinuria, and renal failure [14]. Correspondingly, our patients with composite primary outcomes had higher baseline proteinuria and systolic blood pressure levels. Possibly due to the proteinuria, these patients had lower serum albumin levels than the patients without the composite primary outcome

Wikstad et al. conducted a study on 36 patients with solitary kidneys aged <12 years who underwent unilateral nephrectomy due to unilateral renal agenesis or hydronephrosis. The patients were divided into three groups according to their follow-up times and compared with 23 healthy control patients. In that study, an increase in albuminuria and a decrease in GFR were observed as the follow-up period increased in patients with solitary kidneys. 47% of those patients experienced microalbuminuria and late-onset eGFR decline in long-term follow-up during adulthood, which could indicate a risk for the development of kidney failure. Also, they showed that there was an increase in the existing kidney size in patients with solitary kidneys compared to patients in the control group [16]. In our study, 27 patients whose right kidney was not atrophic and 34 patients whose left kidney was not atrophic underwent kidney measurement with both first and control USG during follow-up. The compensatory response expected in patients with atrophic kidneys due to an atrophic kidney did not occur in our patients. This could be because of the limited number of patients. Besides these, our study was conducted in adult patients (the most common cause of unilateral atrophy was unknown in our cohort. However, it was not possible to conclude that these cases were congenital anomalies, such as kidney hypoplasia). There may be differences between adult and pediatric patients regarding compensatory hypertrophy. In addition, the follow-up time may have affected the compensatory hypertrophy. In our patients, control ultrasonography was not performed according to a specific follow-up protocol, because of the retrospective nature of the study.

Our study has some limitations. First, it was a retrospective study. Due to its retrospective nature, some data were missing (for example, the baseline proteinuria level was present in 60 of 166 patients with at least 3 months of follow-up). Second, the etiology of atrophic kidneys could not be determined in most patients (most of them possibly had congenital atrophic kidneys). Third, there was no data on body mass index, smoking, or cardiovascular diseases that could affect long-term renal outcomes. However, as mentioned before, there are limited numbers of studies on unilateral atrophic kidneys in adults, and our study can contribute to the literature because of the large number of patients with a long follow-up time.

## 5. Conclusions

Baseline serum creatinine and albumin levels were associated with progression to CKD stage 5 in patients with unilateral atrophic kidneys. In addition, renal artery stenosis-related unilateral kidney atrophy might be associated with worse outcomes than other etiologies according to our data. Close follow-up and early nephrology referral of adult patients with unilateral atrophic kidneys should be considered to prevent kidney failure. New prospective studies with larger patient numbers must be conducted to determine the best treatment and follow-up approach for such patients.

As it is known, baseline serum creatinine and albumin levels are risk factors for CKD stage 5 not only in patients with unilateral atrophic kidney but also in patients with bilateral normal kidney size. However, we still wanted to emphasize the impact and importance of these two important criteria in real life for this patient group, to draw attention to this issue not only for nephrologists but also in general medical practice, and to contribute to the literature on this patient group.

## Figures and Tables

**Table 1:** Characteristics of 199 patients with unilateral atrophic kidneys

Demographic and radiological features	n = 199
Female	114 (57. 3 %)
Hypertension, n (%)	133 (66.8 %)
RAS blockade usage, n (%)	93 (46.7 %)
Diabetes mellitus, n (%)	25 (12.6 %)
Atrophic kidney distribution, n (right/left)	103 (51.8 %) / 96 (48.2 %)
Atrophic kidney dimensions (right/left) (mm)	72.8±17.9 / 73.6±18.7
Non-atrophic kidney dimensions (right/left) (mm)	117.9±14.2 / 118.2±14.9
Tc-99m-DMSA ratios (right/left) (n=76/87) (%)	14.7±10.6 / 15.6±12.1

**Table 2:** Etiology of atrophic kidneys

Etiology	n=199
Chronic pyelonephritis, n (%)	34 (17%)
Nephrolithiasis, n (%)	19 (9.5%)
Vesicoureteral reflux, n (%)	7 (3.5%)
Renal artery stenosis, n (%)	6 (3%)
Congenital causes, n (%)	4 (2%)
Others, n (%)	2 (1%)
Unknown, n (%)	127 (63.8%)

**Table 3:** Accompanying urinary system anomalies in patients with atrophic kidney

Urinary system anomalies	n=199
None [n (%)]	189 (95%)
Ectopic kidney [n (%)]	4 (2%)
Cryptoorchidism [n (%)]	2 (1%)
Double collecting system [n (%)]	2 (1%)
Prune-Belly Syndrome [n (%)]	1 (0.5%)
Ureterocele [n (%)]	1 (0.5%)

**Table 4:** Demographics, baseline laboratory and clinical data, and outcomes of 166 patients with at least 3 months of follow-up

Parameters	
Median age at the first presentation (IQR 25-75)	45 (32.5–55)
Gender, female/male (%)	57.8 % / 42.2 %
Atrophic kidney, right/left (%)	51. 8 % / 48.2 %
Atrophic left kidney craniocaudal length, mm (IQR 25–75)	72 (63–84.5)
Atrophic right kidney craniocaudal length, mm (IQR 25–75)	75.5 (62.75–87.25)
Atrophic left kidney DMSA, percentage (IQR 25–75) (n=74)	14 (6–27)
Atrophic right kidney DMSA, percentage (IQR 25–75) ( n=63)	14 (5.7–21)
Mean systolic blood pressure, mmHg (± SD)	134.3 ± 28.2
Mean diastolic blood pressure, mmHg (± SD)	83.3 ± 16.4
Accompanying urinary anomalies	12 (7.2 %)
Family history of kidney disease, n (%)	10 (6 %)
Hypertension, n (%)	113 (68.1 %)
Diabetes mellitus, n (%)	23 (13.9 %)
Median serum creatinine, mg/dL (IQR 25–75)	1 (0.8–1.4)
Median eGFR (CKD-EPI), ml/min/1.73 m^2^ (IQR 25–75)	71 (47.5–103)
Mean serum albumin, g/dL (± SD) (n=153)	4.38 ± 0.45
Median LDL, mg/dL (IQR 25–75) (n=113)	122 (100–151)
Median TG, mg/dL (IQR 25–75) (n=113)	116 (81.5–180.5)
Median total cholesterol, mg/dL (IQR 25–75) (n=113)	201 (168–229.5)
Median HDL, U/L (IQR 25–75) (n=113)	47 (37–56)
Median proteinuria, g/g or g/day (IQR 25–75) (n=60)	0.07 (0.16–1)
Hematuria (>3 per HPF), n (%) (n=140)	33 (19.9 %)
Pyuria (>5 per HPF, n (%) (n=140)	37 (22.3 %)
Median follow-up time, months (IQR 25–75)	69.5 (37–139)
RAS blockade (ACEi or ARB), n (%)	83 (50 %)
Median follow-up time, months (IQR 25–75)	69.5 (37–139)
CKD stage 5, n (%)	15 (9 %)
Doubling of serum creatinine, n (%)	13 (7.8 %)
Composite primary outcome, n (%)	19 (11.4 %)
Nephrectomy requirement, n (%)	6 (3.6 %)
New-onset proteinuria, n (%)	17 (10.2 %)
> % 50 increased rate of proteinuria level according to baseline, n (%)	4 (2.4 %)
Mortality, n (%)	6 (3.6 %)

**Table 5:** Demographics, baseline laboratory, and clinical data of 166 patients with and without composite primary outcome

Parameters	Without outcome (n=147)	With outcome (n=19)	P value
Median age at the first presentation (IQR 25–75)	45 (31–55)	37 (34–56)	0.971
Gender, female/male (%)	86/61	10/9	0.626
Atrophic kidney, right/left (%)	73/74	13/6	0.124
Atrophic left kidney craniocaudal length, mm (IQR 25–75)	70.5 (60.5–82.75)	81 (70–92)	0.23
Atrophic right kidney craniocaudal length,, mm (IQR 25–75)	75.5 (61.75–88.25)	75.5 (66–85.75)	0.82
**Mean systolic blood pressure, mmHg (± SD)**	**132 ± 26.4**	**151.84 ± 35.4**	**0.004**
Mean diastolic blood pressure, mmHg (± SD)	81.9 ± 14.4	93.94 ± 25.2	0.055
Accompanying urinary anomalies, n (%)	12 (8.2 %)	0 (0 %)	0.364
Family history of kidney disease, n (%)	10 (6.8 %)	0 (0 %)	0.606
**Hypertension, n (%)**	96 (65.3 %)	17 (89.5 %)	**0.033**
Diabetes mellitus, n (%)	23 (15.6 %)	0 (0 %)	0.078
**Median serum creatinine, mg/dL (IQR 25**–**75)**	**1 (0.8**–**1.3)**	**2 (1.2**–**2.6)**	**<0.001**
**Median eGFR (CKD-EPI), ml/min/1.73 m^2^ (IQR 25**–**75)**	**75 (52**–**106.25)**	**40 (24**–**60)**	**<0.001**
**Mean serum albumin, g/dL (± SD)**	**4.42 ± 0.44**	**4.05 ± 0.43**	**0.001**
Median LDL, mg/dL (IQR 25–75)	122 (101–151.75)	126 (90.75–149.5)	0.713
Median TG, mg/dL (IQR 25–75)	116 (78–181)	123 (91.75–181.5)	0.516
Median total cholesterol, mg/dL (IQR 25–75)	196 (169–229)	204 (159.75–242.75)	0.907
Median HDL, U/L (IQR 25–75)	47 (36–56)	46 (40–53)	0.686
**Median proteinuria, g/g or g/day (IQR 25**–**75)**	**0.1 (0.06**–**0.5)**	**2 (0.95**–**2.85)**	**<0.001**
Hematuria (>3 per HPF), n (%)	29/140 (20.7 %)	4/18 (22.2 %)	1.000
Pyuria (>5 per HPF, n (%)	33/140 (23.6 %)	4/18 (22.2 %)	1.000
RAS blockade (ACEi or ARB), n (%)	73 (49.6 %)	10 (52.6 %)	0.807
**Renal artery stenosis**	**3 (2 %)**	**3 (15.8 %)**	**0.021**
Vesicouretheral reflux	4 (2.7 %)	1 (5.3 %)	0.460

**Table 6:** Follow-up data of 166 patients with and without composite primary outcome

Parameters	Without outcome (n=147)	With outcome (n=19)	P value
Median follow-up time, months (IQR 25–75)	72 (37–139)	64 (38–127)	0.835
Recurrent urinary tract infection	42 (28.6 %)	6 (31.6 %)	0.786
Nephrolithiasis	31 (21 %)	3 (15.8 %)	0.767
Nephrectomy requirement, n (%)	5 (3.4 %)	1 (5.3 %)	0.524
New-onset or > % 50 increased rate of proteinuria level according to baseline, n (%)	17/110 (15.5 %)	4/13 (30.8 %)	0.234
Mortality, n (%)	5 (3.4 %)	1 (5.3 %)	0.524

**Table 7:** Complications associated with atrophic kidneys

Complications	n=199
CKD stage 5 [n (%)]	15 (7.5 %)
Doubling of serum creatinine [n (%)]	13 (6.5 %)
Nephrolithiasis [n (%)]	41 (20.6%)
Recurrent urinary tract infections [n (%)]	51 (25.6%)
Nephrectomy [n (%)]	7 (3.5%)

**Table 8:** Multivariate logistic regression analysis for risk factors in primary outcome for 166 patients with at least 3 months of follow-up

	*p*	ODDS	95% CI	

			Lower	Upper
**Baseline serum albumin**	***0.034*** *	0.243	0.66	0.899
**Baseline serum creatinine**	***< 0.001*** *	7.786	0.941	22.956
**Renal artery stenosis (Yes)**	***0.015*** *	12.361	1.626	93.991
**Baseline systolic blood pressure**	***0.332***	1.178	0.151	9.222

* p < 0.05

**Abbreviations in tables:** ACEi; Angiotensin converting enzyme inhibitor, ARB; Angiotensin receptor blocker, CI; Confidence interval, CKD-EPI; Chronic kidney disease epidemiology collaboration, DMSA; Dimercaptosuccinic acid, ESKD; End stage kidney disease, eGFR; estimated glomerular filtration rate, HPF; High power field, IQR; Interquartile range, LDL; Low-density-lipoprotein, SD; Standard deviation, TG; Triglyceride

**Table 9:** Correlation matrix for the variables in multivariate logistic regression analysis for 166 patients with at least 3 months of follow-up

Correlation Matrix						
		Constant	Baseline Creatinine (mg/dL)	Baseline Systolic BP (mmHg)	RAS(1)	Baseline Albumin (gr/dl)
Step1	Constant	1.000	−0.307	−0.543	0.407	−0.839
	Baseline Creatinine (mg/dL)	−0.307	1.000	0.055	0.157	0.069
	Baseline Systolic BP (mmHg)	−0.543	0.055	1.000	−0.354	0.080
	RAS(1)	0.407	0.157	−0.354	1.000	−0.245
	Baseline Albumin (gr/dl)	−0.839	0.069	0.080	−0.245	1.000

**Establishing the multivariate model:** Baseline eGFR, proteinuria, baseline diastolic pressure variables, and the presence of hypertension were not included in the multivariate analysis model because of the high correlations between other variables. Hypertension was not included in the multivariate model because of the strict clinical relationship between systolic and diastolic blood pressure and renal artery stenosis. Diastolic blood pressure significantly correlated with systolic blood pressure (r = 0.841, p < 0.001, Pearson’s test). Therefore, the diastolic blood pressure was not included in the multivariate model. In addition, baseline serum creatinine level and eGFR were significantly correlated (r = −0.927, p < 0.001 with Spearman’s test), and eGFR was excluded from the multivariate model. In addition, proteinuria and serum albumin had high coefficient value (0.735) in the correlation matrix of the multivariate analysis model. Baseline proteinuria levels were present in 60 of the 166 patients. Hence, proteinuria was excluded in the multivariate analysis model. The final multivariate analysis model included baseline creatinine level, baseline serum albumin level, and the presence of renal artery stenosis.
